# A neglected priority? The importance of surgery in tackling global health inequalities

**DOI:** 10.7189/jogh.05.010304

**Published:** 2015-06

**Authors:** Ewan D. Kennedy, Cameron J. Fairfield, Stuart J. Fergusson

**Affiliations:** 1 Medical student, The College of Medicine and Veterinary Medicine, The University of Edinburgh, Edinburgh, Scotland, UK; 2 Specialty registrar in General Surgery, Wishaw General Hospital, Wishaw, Scotland, UK

“*I was thinking that I was already dead… now I can talk with you people, I’m so happy!*” These words, spoken quietly but with great warmth by a young woman devastated by a vesicovaginal fistula and restored to dignity through compassionate operative treatment, cut cleanly through divides of geography, culture, and class. More eloquently than any statistic she speaks of the life–changing and life–saving importance of surgical services in all health care systems. The woman’s testimony features in Jaymie Ang Henry’s film, ‘The Right to Heal’ [1], a deeply moving piece of advocacy that makes a clear case for focusing far greater global attention on the huge need to achieve more equitable access to essential surgery. This is the case we take up here, with a particular focus on the needs of sub–Saharan Africa.

## GLOBAL SURGICAL NEEDS

Every year an estimated 234 million major operations are performed worldwide, yet only 3.5% of procedures are performed in the poorest third of the world’s population [2].

The Disease Control Priorities for Developing Countries Project reported that 11%–15% of the global disease burden is amenable to surgical treatment [3]. This report also estimated the impact of surgical disease through disability–adjusted life years (DALYs) – a metric which combines ‘number of years lived with disability’ with ‘years of life lost’ through death prior to anticipated life expectancy. DALYs were considerably higher in low and middle income countries (LMICs), ranging from a high on the African continent of 38 DALYs per 1000 population to a worldwide low in the Americas of 21 per 1000. However, these figures do not account for a huge range of other surgical diseases.

There is a paucity of patient–level data on surgical outcomes in LMICs [4], and it is likely that LMICs are afflicted both by poor access to surgical services and the highest levels of adverse surgical outcomes. Access to essential surgical care, taken for granted in developed health care systems, remains unavailable to many of the world’s poorest [2,5].

## SURGICAL CARE PROVISION IN SUB–SAHARAN AFRICA

**Figure 1:**
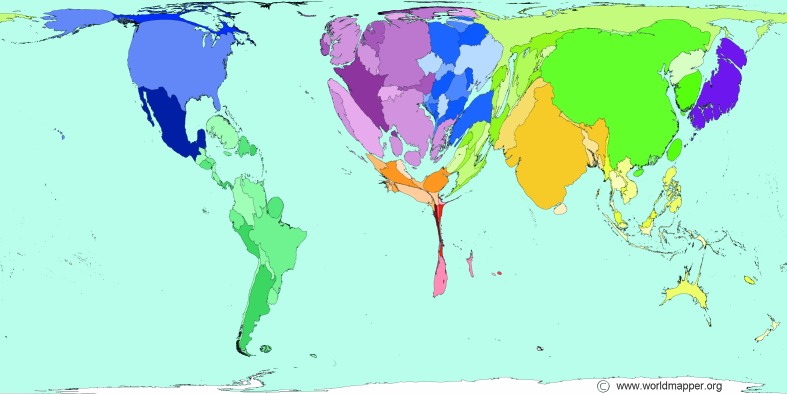
Proportionate distribution of doctors worldwide. Source: Worldmapper.org [6], Creative Commons Licence for this image.

Little definitive knowledge about volume and availability of surgical care in sub–Saharan Africa exists as most evidence is anecdotal. Estimates suggest the burden of unmet need is vast [2]. **Figure 1** shows a representation of world territory size proportional to the global medical workforce located in that area, vividly demonstrating the region’s profound shortage of doctors in general.

**Figure 2:**
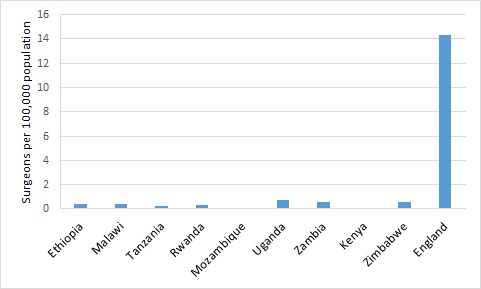
Number of surgeons per capita in selected countries. Source: The Royal College of Surgeons of England and the College of Surgeons of East Central and Southern Africa (COSECSA) [7,8].

This shortage is particularly marked with regard to surgeons. **Figure 2** illustrates the low number of surgeons per head of population in select African nations. Surgical specialists are in particularly short supply: Nigeria had 1 paediatric surgeon per 2.2 million children in 2003 [9]. Surgeons are also inequitably distributed throughout sub–Saharan African countries: 80%–90% of surgeons work in urban areas, although 85% of the population live in rural regions [7]. This situation arises from unattractive rural conditions such as poor working environment, lack of transport, limited career progression, lack of exposure to surgical techniques and the migration of other health care workers. Doctor migration, or ‘brain drain’, is a huge problem in sub–Saharan Africa.

The lack of human resources, infrastructure and facilities means the need for surgical services is immense. Most district hospital operating theatres in Malawi do not have dedicated staff and half lack adequate instruments, including sutures, for common surgical procedures [10]. In Chad, Madagascar, Niger, Burkina Faso and Ethiopia, Caesarean–sections only account for 0.4–1.0% of all births, while the general consensus for an ideal global Caesarean section rate is 10–15% [5]. This suggests that this life–saving procedure is not available to most expectant women, and must be a key reason why women in sub–Saharan Africa have an adult lifetime maternal mortality risk of 1 in 38, compared with 1 in 3700 in developed countries [11].

## SURGERY AND GLOBAL HEALTH PRIORITIES

The global health successes of the last thirty years have been largely against infectious diseases. Public health organisations are approaching eradication of polio and smallpox, and mortality from HIV/AIDS, tuberculosis and malaria are declining due to well–established interventions [12]. Largely through LMICs industrialisation, improved health care systems and the successes of the global drive to eradicate infectious disease, the usual trends of disease have been altered and non–communicable diseases now surpass infectious diseases as leading contributors to morbidity and mortality [13].

Surgery has a neglected profile in global health, taking a back seat to other priorities despite the fact that surgical diseases disproportionately affect the world’s poorest people [13]. Arguably, essential surgical care should be part of the basic right to health care. Surgery will need to assume a more prominent role in public health as the balance is tipped toward an increasing prevalence of surgical conditions.

There is no specific mention of surgery in the Millennium Development Goals (MDGs) despite the burden of surgically–amenable disease. Additionally there has been little mention of how improving basic surgical care would help to achieve targets within the MDGs especially MDG4 (reduce child mortality), MDG5 (improve maternal health) and MDG6 (combat HIV/AIDS) [14]. Traditionally surgery has been viewed as an expensive tool of last resort after a failure of medical therapy and one that has limited value as a prophylactic intervention.

**Figure:**
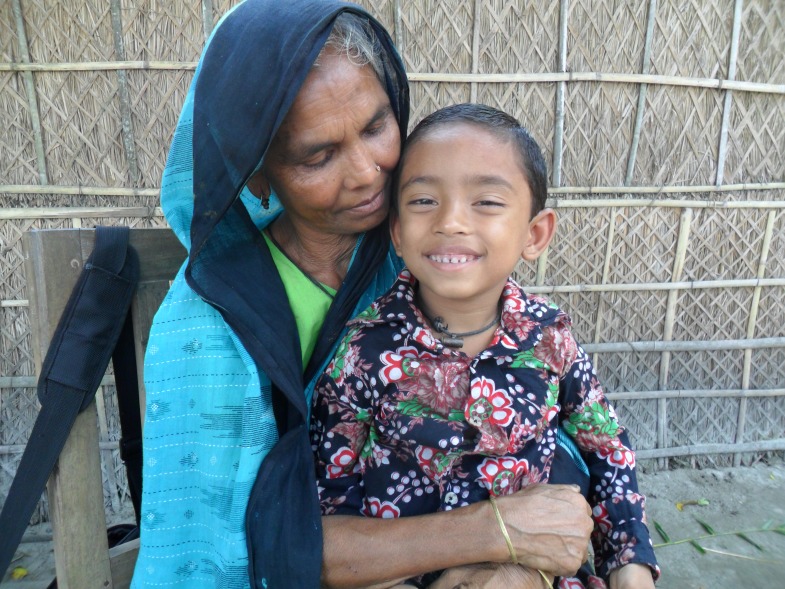
Photo: Child treated for congenital cataract (Courtesy of Orbis; http://gbr.orbis.org/news/entry/simul-can-play; used with permission)

## THE CASE FOR PRIORITISING SURGICAL CARE

The existence of profound gaps in surgical provision within sub–Saharan Africa may not be surprising, but the case for focusing on surgical care development may not be immediately obvious in the context of so much unmet need. We put forward two important arguments.

### Cost–effectiveness

Recent cost–effectiveness studies [15] refute the popular attitude that surgery is an unaffordable financial expense in the developing world. They demonstrate that simple and safe surgery at district hospitals represents a cost–effective component of health care which not only transforms the life of an individual; rather it also has the capacity to empower communities to enter work and support the local economy by preventing disability.

Surgical treatment of cataract provides an excellent example of the role that surgery can play in restoration of livelihoods. After living for two years with congenital cataract, the young child pictured in the illustration was granted the gift of clear sight through the charity Orbis following an operation. This enabled him to play with friends and attend school for the first time allowing him to receive an education.

This simple and effective surgery provides a child the possibility of a future free from the disabling condition and also frees another individual who would likely have been involved in their care. The link between surgical intervention in cataract and improved quality of life as well as reduction in poverty in LMICs is demonstrable through large studies. One study [16] showed surgical treatment of cataract leads to sustained improvement in per capita expenditure of the household of the patient for up to 6 years. In the Philippines, per capita expenditure increased from US$ 22 to US$ 39 per person per month in those receiving curative surgery compared to an increase of US$ 29 to US$ 37 in healthy controls.

**Figure 3:**
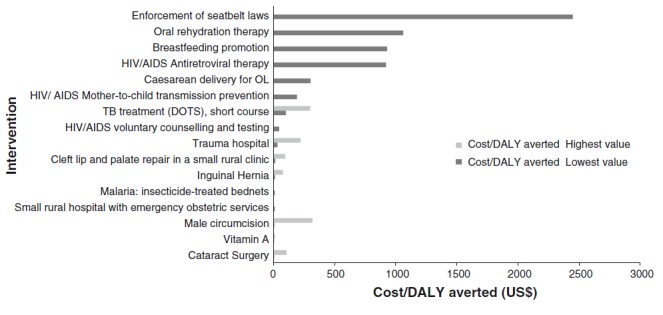
The cost–effectiveness of surgical and non–surgical public health interventions. Source: Grimes et al. [15], used with permission.

Data from Grimes et al [15] demonstrate that the cost–effectiveness of surgical interventions compares well with other public health measures (**Figure 3**) and makes a powerful case that their utility has been unfairly neglected.

### Wider health benefits

An improvement in surgical care would have wider benefits on health problems beyond specifically surgical disease. Basic surgical provision provides a valuable adjunct to the medical and social therapies already adopted by proponents of MDGs 4, 5 and 6 [14] as mentioned above.

With regard to MDG6 (combating HIV/AIDS), improving surgical provision would give clear support to this objective. As little as 18% of surgical centres in LMICs provide appropriate eye protection and 48% provide a sharps bin for surgical staff during surgical procedures [14]. Basic training and provision of protective equipment would likely reduce the rates of HIV transmission. Similarly, despite evidence to support Caesarean sections in HIV–positive mothers, 54 countries have Caesarean section rates under 10% [5]. The majority of these countries are in Africa where HIV rates are extremely high ranging from 1.3–12% per region [12]. Striving to increase the availability of Caesarean sections in these areas will be a useful tool in the control of HIV.

## THE WAY AHEAD

The world can no longer afford to neglect the importance of surgical services. How then does the global community tackle the immense global inequalities in surgical care? We offer some evidence–based suggestions.

### Personnel

In view of the profound manpower shortages outlined above, a key goal for all LMICs is to increase the size and skill of their surgical workforce, and to distribute that workforce more equitably within countries. Appropriate remuneration and quality specialist training are important to keep doctors engaged. There are many successful training schemes in LMICs which have been established to equip national surgeons to meet local needs [17].

Nevertheless, the acute shortage of medical staff has meant that a central plank in the health care delivery strategy of many LMICs has been the development of a cadre of paramedical professionals, who in some countries undertake the majority of surgical procedures, particularly in smaller district hospitals, with good results [18]. Their professional development should continue to be supported.

### Infrastructure and equipment

Equipment and infrastructure gaps in LMICs are well documented [14] and efforts to improve global surgical provision must include efforts to address this problem. In a recent convenience sample of 70 hospitals across 7 LMICs [19], only 59% of hospitals had a pulse oximeter in every theatre, with 33% having a pulse oximeter in recovery facilities.

Well–meaning but inappropriate donations can be unhelpful, and so gifts of equipment must be carefully planned and evaluated. Thoughtfully planned interventions such as the Lifebox Foundation’s supply of purpose–built pulse oximeters to LMIC settings, with associated training, provide an example of the effectiveness of best practice in this area [20].

### Academic activity

One of the activities which has undergirded success in other areas of health care development has been focused academic work. Although there are many health research institutions in LMICs, the volume and influence of their activities needs significant further improvement [4]. International partnerships and collaborative work such as the GlobalSurg study of emergency abdominal surgery outcomes [21] can be powerful drivers of a strong local audit culture that needs developed.

In the age of the internet, partners have the opportunity to bring high–quality academic training directly to surgeons working within LMICs which will contribute to the effort to avoid ‘brain drain’ [22].

### Advocacy

Finally, achieving greater focus on surgery as a developmental priority will also require political engagement [23], something that surgeons find difficult given their overwhelming clinical workload. This will mean advancing international collaborations such as the World Health Organization’s Global Initiative for Emergency and Essential Surgical Care [24], and reframing some surgical needs in terms of current political priorities, such as the 2015 Millennium Development Goals [14].

With a comprehensive plan of engagement, surgery can be redeemed from its “neglected stepchild” [25] status in global health, with huge benefits to the global population.
